# Sustained virological response halts fibrosis progression: A long-term follow-up study of people with chronic hepatitis C infection

**DOI:** 10.1371/journal.pone.0185609

**Published:** 2017-10-24

**Authors:** Swee Lin G. Chen Yi Mei, Alexander J. Thompson, Britt Christensen, Georgina Cunningham, Lucy McDonald, Sally Bell, David Iser, Tin Nguyen, Paul V. Desmond

**Affiliations:** 1 Department of Gastroenterology, St Vincent’s Hospital, Melbourne, Australia; 2 Department of Medicine, University of Melbourne, Melbourne, Australia; National Taiwan University Hospital, TAIWAN

## Abstract

**Background/Aims:**

Long-term follow-up studies validating the clinical benefit of sustained virological response (SVR) in people with chronic hepatitis C (CHC) infection are lacking. Our aim was to identify rates and predictors of liver fibrosis progression in a large, well characterized cohort of CHC patients in whom paired liver fibrosis assessments were performed more than 10 years apart.

**Methods:**

CHC patients who had undergone a baseline liver biopsy pre-2004 and a follow up liver fibrosis assessment more than 10 years later (biopsy or liver stiffness measurement (LSM) using transient elastography [FibroScan]) were identified. Subjects who had undergone a baseline liver biopsy but had no follow up fibrosis assessment were recalled for LSM. Fibrosis was categorised as mild-moderate (METAVIR F0-2 / LSM result of *≤* 9.5 kPa) or advanced (METAVIR F3-4/ LSM >9.5 kPa). The primary objective was to assess the association between SVR and the rate of liver fibrosis progression over at least 10 years, defined as an increase from mild-moderate fibrosis at baseline liver biopsy (METAVIR F0-2) to advanced fibrosis at follow-up liver fibrosis assessment.

**Results:**

131 subjects were included in this analysis: 69% male, 82% Caucasian, 60% G1 HCV, 25% G3 HCV. The median age at F/U fibrosis staging was 57 (IQR 54–62) years with median estimated duration of infection 33-years (IQR 29–38). At F/U, liver fibrosis assessment was performed by LSM in 86% and liver biopsy in 14%. The median period between fibrosis assessments was 14-years (IQR 12–17). 109 (83%) participants had received interferon-based antiviral therapy. 40% attained SVR. At F/U, there was a significant increase in the proportion of subjects with advanced liver fibrosis: 27% at baseline vs. 46% at F/U (p = 0.002). The prevalence of advanced fibrosis did not change among subjects who attained SVR, 30% at B/L vs 25% at F/U (p = 0.343). However, advanced fibrosis became more common at F/U among subjects with persistent viremia: 10% at B/L vs 31% at F/U (p = 0.0001). SVR was independently associated with protection from liver fibrosis progression after adjustment for other variables including baseline ALT (p = 0.011), duration of HCV infection and mode of acquisition.

**Conclusion:**

HCV eradication is associated with lower rates of liver fibrosis progression. The data support early treatment to prevent long-term liver complications of HCV infection.

## Introduction

Approximately 115 million people are chronically infected with hepatitis C (HCV) worldwide, including 230,000 Australians.[1,2] HCV is associated with complications including liver cirrhosis, liver failure and hepatocellular carcinoma. HCV is the most common indication for liver transplant in high-income countries including Australia. Antiviral therapy can eradicate HCV. It is assumed that eradicating HCV will prevent these complications.

The landscape of HCV treatment has changed dramatically over recent years. Interferon-free combinations of direct acting antiviral (DAA) agents are now standard of care, with cure rates approaching 100% and excellent tolerability. However the cost associated with the new DAAs is a major barrier to treatment access. Therefore, it is important to evaluate long-term clinical outcomes, to confirm that viral eradication is associated with predicted clinical benefit.

Clinical trials evaluating antiviral therapy for HCV use sustained virological response (SVR) as a surrogate for clinical benefit. Until recently, there has been little data available confirming the impact of SVR on long-term clinical outcomes of people with CHC. In particular, there are limited data concerning the long-term benefit of SVR on fibrosis progression in non-cirrhotic subjects.

In selected high-risk populations of subjects with advanced fibrosis or cirrhosis, SVR is associated with improved overall and liver-related survival, as well as reduced rates of hepatic de-compensation and hepatocellular carcinoma.[3–7] These results have been replicated in numerous, multi-centre studies of up to 500 subjects with a follow up of 3–8 years. However, data demonstrating a clinical benefit from SVR in people with less advanced disease are scarce.

There are data to suggest that SVR leads to fibrosis regression. Clinical trials based on paired liver biopsies have shown SVR to be associated with histological improvement in both grade and stage. However, these studies were limited by a short-term follow up of only 12–24 months.[8–10] Recent data from Spain suggest that SVR is associated with significant reduction in the risk of progression to cirrhosis in a population of patients with median duration of follow-up of 10 years. A similar retrospective study in Korea observed an association between SVR and lower risk of cirrhosis over median follow-up of 4 years. In both studies, the definition of cirrhosis at long-term F/U was based on radiological features for most patients. Transient elastography has greater sensitivity for the presence of advanced fibrosis / cirrhosis than ultrasound, meaning that these studies may have under-estimated the risk of fibrosis progression.

We describe a large, well-characterized cohort of CHC patients followed longitudinally in whom paired liver fibrosis assessments were performed more than 10 years apart. We used transient elastography and liver stiffness measurement to define the presence of advanced fibrosis / cirrhosis at follow-up. We show that achievement of SVR halts liver fibrosis progression. The data supports early treatment of all people with CHC, regardless of liver fibrosis stage, to prevent long-term liver sequelae.

## Methods

### Data resource

The St Vincent’s Hospital Hepatitis C electronic database (HCV database) is a hospital-based patient database that comprehensively records up-to-date patient characteristics (demographic and biochemical), treatment history, liver fibrosis stage and hospital clinic attendance.

### Study population

This was a retrospective / prospective study. Subjects who had undergone a first time liver biopsy before 2004 were identified from the HCV database. For inclusion in the current study, subjects were required to have a second liver fibrosis assessment ≥ 10 years after the original assessment. 106 patients in the database had paired liver fibrosis assessments more than 10 years apart. If follow-up liver fibrosis staging had not been performed more than 10-years after baseline liver biopsy, subjects received an invitation by mail to present for liver stiffness measurement (LSM) and a blood test. 25 subjects were recalled by this method. We excluded subjects co-infected with hepatitis B or HIV. Demographic, clinical, histological and biochemical data were collected from the HCV database.

### Timepoints

CHC was defined as persistent HCV RNA for at least 6-months after onset of acute infection or known exposure. The estimated duration of infection was calculated from the year of known exposure or episode of acute infection. A liver biopsy performed after diagnosis of chronic HCV infection formed the first liver fibrosis assessment. Second liver fibrosis measurement was defined as either a liver biopsy or LSM performed ≥ 10 years after first liver fibrosis assessment. HCV treatment was recorded as the completion date of antiviral therapy, with a sustained virological response (SVR) defined as an undetectable HCV RNA 24-weeks post antiviral therapy.

### Blood test

Blood test included full blood count, electrolytes, liver function test, IL28B genotype, HCV viral load and genotype, and HBV serology.

### Liver fibrosis evaluation and categorisation

Fibrosis was categorised as mild-moderate for a biopsy with a METAVIR stage of 0–2 or a LSM result of *≤* 9.5 kPa and as advanced for a biopsy with a METAVIR stage of *≥* 3 or a LSM result of > 9.5 kPa.

### Liver stiffness measurement by transient elastography

LSM was assessed using transient elastography according to the manufacturer’s guidelines, and considered valid if a minimum of 10 measurements were obtained with at least a 60% success rate and an interquartile range <0.3 of the median value. Operators at our institution had performed a minimum of 100 procedures.

### Liver histology

Liver histology was assessed by pathologists specialized in liver disease. Specimens were obtained by percutaneous liver biopsy, then fixed, paraffin-embedded, and stained with haematoxylin-eosin and Masson’s trichrome. Histological fibrosis stage was scored according to the METAVIR classification, defined as F0, no fibrosis; F1, portal fibrosis without septa; F2, portal fibrosis and few septa; F3, numerous septa without cirrhosis; and F4, cirrhosis.[11]

### Outcomes analysis

The primary objective was to assess the effect of SVR on the rate of liver fibrosis progression over 10-years. Participants with advanced fibrosis (F3/F4), or cirrhosis (F4), at baseline were excluded in this analysis. SVR was defined as an undetectable HCV PCR 6-months post antiviral therapy. Fibrosis progression was defined as an increase from mild-moderate fibrosis at baseline liver biopsy (METAVIR F0-2) to advanced fibrosis at follow-up liver biopsy (METAVIR F3-4) or LSM (>9.5 kPa). The secondary objective was to identify variables influencing liver fibrosis progression, and included age, gender, hepatitis C genotype (genotype 1 vs. non-genotype 1), mode of HCV acquisition (blood transfusion vs. other), body mass index (BMI), baseline ALT, HCV viral load, IL28B genotype (rs12979860) (CC vs. non-CC), estimated duration of infection and SVR. Indirect fibrosis progression rate was defined as the ratio between the METAVIR fibrosis score at baseline liver biopsy and the estimated duration of infection. LSM measurements were converted to corresponding METAVIR fibrosis stage as previously validated.[12–18]

### Statistical analysis

Participant characteristics were presented as median and inter-quartile ranges (IQR) for continuous variables and number (%) for dichotomous variables. McNemar’s test was used to assess for liver fibrosis progression. In the analysis of variables that were associated with liver fibrosis progression, Fisher’s exact test / Chi square test was used for dichotomous variables and Mann-Whitney for continuous variables. The probability level was set at p ≤ 0.05. Variables that were associated with liver fibrosis progression with a p-value < 0.1 were included in multivariable logistic regression models. Statistical analysis was performed using PRISM v6.0.

The study protocol was approved by the Human Research Ethics Committee at St Vincent’s Hospital and conducted according to the Declaration of Helsinki and ICH/GCP guidelines. Written informed consent was obtained for participants recalled for LSMs. Informed consent was not deemed necessary for subjects derived from the HCV database, given the retrospective nature of this study.

## Results

### Study population

131 subjects were included in this analysis. All subjects had had a liver biopsy at our institution prior to 2004. 18 subjects had paired liver histology available more than 10 years apart, 88 subjects had a liver biopsy with a follow-up LSM, and 25 subjects were actively recalled to present for a follow-up LSM and blood test. Of the 106 subjects who did not require recall, 7 did not have blood test results at the time of follow-up fibrosis staging.

Clinical characteristics of the cohort at baseline and follow up fibrosis assessment are summarized in Tables 1 and 2, respectively. 69% (91/131) were male, 82% were Caucasian (108/131) and the median age at follow-up assessment was 57 (IQR 54–62) years. As noted, liver fibrosis assessment at F/U was performed by transient elastography in 86% (113/131) and 14% (18/131) had a repeat liver biopsy. The median period between first and second liver fibrosis assessments was 14-years (IQR 12–17 years). The majority of our cohort (60%, n = 79) was infected with Genotype 1 HCV, followed by Genotype 3 HCV (25%, n = 33). At baseline, 79% (104/131) had mild-moderate liver fibrosis (F0 31% (41/131), F1 33% (43/131), F2 15% (20/131)), and 21% advanced fibrosis (F3 8% (10/131), F4 13% (17/131)). 109 (83%) participants received antiviral therapy. 25% (27/109) were treated with standard interferon-α, 35% (38/109) with standard interferon-α and ribavirin, 33% (36/109) with pegylated interferon-α and ribavirin (PR), 6% (7/109) with a protease inhibitor in combination with (PR) and one participant with an interferon-free combination of DAAs. 40% (44/109) attained SVR. HCC developed in 3 participants during follow up. There were no reports of hepatic de-compensation or death at follow up.

**Table 1:** Participant characteristics at baseline.

Participant Characteristics at Baseline (n = 131)	Median (IQR) or percentage (n)
Age (years)	57 (IQR 54–62)
Male	69% (n = 91)
Ethnicity	82% Caucasian (108)
Age of HCV acquisition (years), n = 101	20 (IQR 17–24)
Estimated duration of infection at time of baseline liver biopsy (years)	17 (IQR 14–22)
Indirect Fibrosis Progression Rate (METAVIR Fibrosis stage/years of infection)	0.05 (IQR 0–0.16)
Patients treated for HCV	83% (n = 109)
Patients attaining SVR during study	40% (n = 44)
Initial ALT (U/L)	94 (IQR 60–152)
Baseline HCV viral load (log_10_ IU/mL)	6.10 (IQR 5.52–6.38)
Liver Fibrosis at baseline	
F0	41 (31%)
F1	43 (33%)
F2	20 (15%)
F3	10 (8%)
F4	17 (13%)
Mode of HCV acquisition	
IVDU	41 (31%)
Blood transfusion	22 (17%)
Tattoo	4 (3%)
Multiple risk factors	37 (28%)
No risk factors	21 (16%)
Unknown	3 (2%)
HCV genotype	
Genotype 1	79 (60%)
Genotype 2	7 (5%)
Genotype 3	33 (25%)
Genotype 4	2 (2%)
Genotype 6	1 (1%)
Unknown	9 (7%)

**Table 2:** Participant characteristics at follow-up fibrosis assessment.

Participant Characteristics at Follow-up Fibrosis Assessment (Liver Biopsy or LSM)	Median (IQR)
Estimated duration of HCV infection at follow-up fibrosis assessment (years), n = 101	33 (IQR 29–38)
Time between Liver biopsy and follow-up fibrosis assessment (years)	14 (IQR 12–17)
BMI	25.2 (IQR 23.5–28.3)
ALT (U/L)	48 (IQR 27–94)

### Liver fibrosis progression secondary to hepatitis C infection

**Fig 1:**
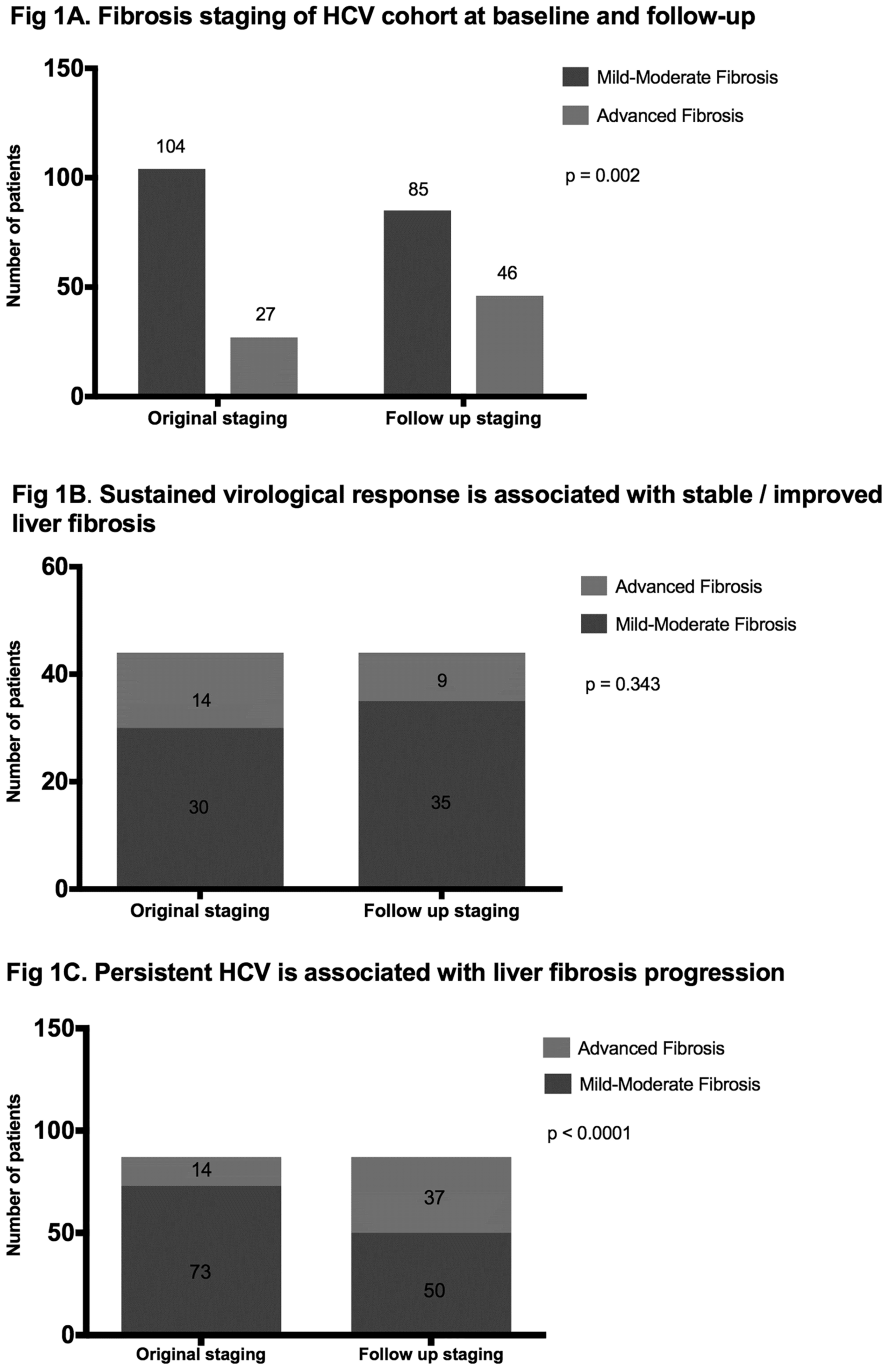
A. Fibrosis staging of HCV cohort at baseline and follow-up. B. Sustained virological response is associated with stable / improved liver fibrosis. C. Persistent HCV is associated with liver fibrosis progression.

At baseline liver biopsy, mild-moderate liver fibrosis (F0-2) was detected in 79% (n = 104) and advanced fibrosis (F3-4) detected in 21% (n = 27). At follow-up, there was a statistically significant increase in the proportion of subjects with advanced liver fibrosis (35% (n = 46), p-value = 0.002, McNemar’s test for comparison of baseline vs. 10 year F/U, (Fig 1A). 21% (n = 27) originally staged as mild-moderate fibrosis had progressed to advanced fibrosis after a median of 14 (IQR 12–17) years. 73% (n = 95) had no change in fibrosis stage, of which 59% (n = 77) and 15% (n = 19) had mild-moderate fibrosis and advanced fibrosis at baseline and follow up, respectively. Among patients with advanced fibrosis at baseline (n = 27), regression to mild-moderate liver fibrosis was observed in 6% (n = 8 (30%)). 7 had received curative HCV treatment. One participant demonstrated fibrosis regression (METAVIR F3 to LSM 5.8kPa after 17-years) despite relapsing to a course of pegylated interferon-a/ribavirin. Of note, they presented with a very low HCV viral load at baseline, with no hepatic inflammation at follow up (ALT 11 U/L).

114 subjects presented with METAVIR F0-F3 at baseline, of which 24% (n = 27) progressed to cirrhosis (F4 or LSM ≥ 13.5 kPa) over 17 years (IQR 12–16).

### Predictors of liver fibrosis progression

#### Sustained virological response following HCV treatment

We considered the association between SVR and liver fibrosis progression. First, we separated patients into those who achieved SVR, and those who did not achieve SVR (Fig 1B and 1c). 83% (n = 109/131) of our cohort received HCV treatment and 40% (n = 44/109) achieved SVR. No significant change in liver fibrosis was observed over a median of 13.5 (IQR 11.5–16.9) years in subjects who were cured of HCV, p = 0.343 (Fig 1B), with fibrosis progression observed in only 3 subjects despite SVR. All had evidence of F2 fibrosis at baseline. One subject had biopsy proven F4 fibrosis at follow up, but required two courses of antiviral therapy (standard IFN and pegIFN/RBV) to achieve SVR. The remaining two subjects progressed to biopsy proven F3 fibrosis, both acquiring HCV secondary to IDU with previous exposure to HBV infection. Alcohol and diabetes were additional co-factors present in one subject. In contrast, 26% (n = 23/87) of subjects who did not receive antiviral therapy or achieve a sustained virological response to treatment, progressed from mild-moderate to advanced fibrosis over a median of 13.7 (IQR 12.3–16.0) years, p = 0.0001, McNemar’s Test (Fig 1C).

A subset analysis was performed on participants who had baseline liver biopsy and follow up LSM (n = 114). This excluded those who had paired liver histology (n = 18). Similar to the findings of the overall cohort, there was a significant increase in the proportion of subjects (32%(n = 36) with advanced fibrosis at follow up, p = 0.038, McNemar’s test for comparison of baseline vs. 10 year f/up (Panel A in S1 Fig). In those who attained SVR (n = 36), liver fibrosis regression occurred after 10-years, p = 0.013 (Panel B in S1 Fig), where as in those who did not attain SVR (n = 62), fibrosis progression was evident at follow-up, p = 0.0001 (Panel C in S1 Fig).

#### Failure to attain SVR and longer estimated duration of infection before HCV treatment initiation predict liver fibrosis progression

To adjust for potential confounding variables, we then considered the association between SVR and liver fibrosis progression using logistic regression analysis. The primary analysis included only those subjects with mild-moderate liver fibrosis at baseline. On univariable analysis, predictors of liver fibrosis progression among those presenting with mild-moderate fibrosis at baseline (n = 104) were high baseline ALT (p = 0.011), longer estimated duration of HCV infection prior to liver biopsy (p = 0.029), longer estimated duration of infection before treatment initiation (p = 0.008) and mode of acquisition (p = 0.002) [Table 3]. On multivariable analysis, SVR was independently associated with protection from liver fibrosis progression (p = 0.001). Shorter duration of infection prior to HCV treatment initiation was also associated with protection from liver fibrosis progression (p = 0.005).

**Table 3:** Predictors of liver fibrosis progression—Failure to attain SVR and longer duration of infection prior to HCV treatment.

Co-variate	No Fibrosis progression	Fibrosis progression	*P*-value	Multivariable logistic regression (*P*-value)
*n*	76	28		
Age of patient (yrs)	56 (IQR 53–62)	60 (IQR 56–63)	0.188	
Gender	55 (72%)	18 (64%)	0.472	
HCV genotype (G1 vs. other)	47 (62%)	16 (57%)	0.351	
HCV acquisition (blood transfusion vs. other)	17 (22%)	16 (57%)	**0.002**	0.412
Estimated duration of HCV infection till liver biopsy (years)	16.5 (IQR 12.0–21.5)	19.0 (IQR 15.0–28.0)	**0.029**	0.318
Fibrosis rate to original liver biopsy (METAVIR stage/years of infection)	0.0387 (IQR 0–0.656)	0.0253 (IQR 0–0.0804)	0.984	
Age of acquisition	20 (IQR 17–22)	21 (IQR 15–25)	0.973	
Failure to attain SVR	47 (62%)	23 (82%)	**0.060**	**0.001**
Baseline ALT (U/L)	87 (IQR 55–121)	125 (IQR 76–175)	**0.011**	0.493
Baseline Viral Load (IU/mL)	891012 (IQR 344980–2405270)	1455260 (IQR 1148700–1744878)	0.521	
Baseline ferritin	226 (IQR 132–320)	273 (IQR 147–596)	0.170	
Baseline AFP	5 (IQR 5–6)	5 (IQR 1–7)	0.469	
Caucasian vs. other	62 (82%)	24 (86%)	0.774	
Estimated duration of infection before treatment initiation (years)	20.0 (IQR 15.0–28.0)	28.5(IQR 24.0–34.3)	**0.008**	**0.005**

A separate analysis was performed to include subjects presenting with METAVIR F0 –F3 (n = 114) at baseline. Predictors of fibrosis progression on univariable analysis included longer estimated duration of infection prior to liver biopsy, failure to achieve SVR, high baseline ALT, high baseline ferritin and longer estimated duration before treatment initiation. Similar to the previous findings, attainment of SVR and shorter duration of HCV infection prior to treatment initiation were also associated with protection from liver fibrosis progression on multivariable analysis (see S1 Table).

#### Longer estimated duration of infection prior to HCV treatment initiation predicts liver fibrosis progression in HCV treatment failure

When we considered the population of participants who did not achieve SVR and had mild-moderate fibrosis at baseline (n = 57), the independent predictors of liver fibrosis progression were mode of acquisition, high baseline ferritin, and longer estimated duration of infection prior to HCV treatment initiation (see S2 Table). On multivariable analysis, shorter duration of infection prior to HCV treatment was associated with protection from liver fibrosis progression (p = 0.0002). This was also a significant predictor among participants who were untreated or did not attain SVR (p = 0.032) (see S3 Table).

## Discussion

In this well-characterised cohort of patients followed longitudinally for more than 10 years, we have shown that SVR is associated with a reduction in the risk of liver fibrosis progression. Fibrosis progression occurred in only 7% of those who achieved SVR in comparison to 30% of non-responders to antiviral therapy, over a decade. The three participants who did develop fibrosis progression despite SVR were noted to have additional co-factors such as alcohol and diabetes. This highlights the importance of early treatment initiation, as well as aggressive management of co-factors to avoid fibrosis progression.

Alongside SVR, early treatment initiation was found to be independently associated with protection against liver fibrosis progression. This was evident in both the overall cohort [20-yrs (IQR 15.0–28) vs. 29-yrs (IQR 24–35), p 0.005)], and among those who failed HCV treatment (see supplementary data). The importance of early curative therapy is underscored by the poor prognosis of individuals with advanced fibrosis/cirrhosis and failed HCV treatment. This particular cohort of patients is well described in the literature, most recently in a retrospective analysis of four hundred participants that observed disease progression and death to occur in 42% and 25% respectively, within a decade.[19]

Our results support the findings of two recent retrospective analyses with data to suggest that SVR significantly reduces the long-term risk of liver fibrosis progression. One study from Korea followed two hundred and eighty subjects for 4-years, of which 80% had received antiviral therapy. A lower incidence of cirrhosis was reported among those who attained SVR than without (0.6% vs. 33.9%, p = 0.001). A large study from Spain (n = 1289) also reported a lower incidence of cirrhosis with SVR after a 12-year follow-up period (2.2% vs. 28% of non-responders).[20] Of note, these studies included radiological findings, a less sensitive test than transient elastography, to define cirrhosis.

Fibrosis progression occurred in 21% of our cohort after a 14-year follow-up period, with the majority (n = 16, 59%) developing cirrhosis (Metavir F4 or LSM ≥ 13.5kPa). The cirrhosis rate at follow-up was 21%. This is consistent with previous estimates. A systematic review of 57 studies estimated progression to cirrhosis to be 21% at 20 years among liver clinic attendees.[21] More recently, a UK study found rates of cirrhosis of 23% at 20 years among individuals referred to a tertiary referral centre.[22] However, an Australian series of four hundred and fifty-five participants attending liver clinic reported a cirrhosis prevalence of 12% after 12 years (IQR 7-17yrs).[23] All of these studies formed estimates based on a single liver biopsy and an estimated date of infection, and may have underestimated the rate of HCV-related fibrosis progression in the liver-clinic setting.

Our results contribute to the growing body of evidence that curative HCV treatment prevents long-term fibrosis progression, and supports the notion that access to DAA therapy should be made widely available to all individuals regardless of fibrosis stage.

There are number of limitations to our study. Participants were identified from a HCV database, and although this database was inclusive of all HCV-infected attendees at our tertiary hospital, it is possible selection bias may have occurred. Furthermore, our patient population may be overrepresented by more severe illness and rapid disease progression, compared to a community-based cohort. We were not able to collect detailed data concerning [24–27] behavioural factors that have been associated with the natural history of CHC, including alcohol consumption, marijuana use and coffee consumption, were not included in our analysis.[28,29] Liver inflammation has been shown to alter LSMs and we potentially may have over estimated the degree of fibrosis in participants with high levels of ALT. However, fluctuating levels of inflammation / ALT are more characteristic of chronic hepatitis B infection and a study of one hundred and fifty HCV subjects who underwent a liver biopsy and LSM, showed no impact of ALT on LSM.[30] Despite these limitations, we believe the clinical message is strong, and the cohort is unique for the duration of long-term follow-up with paired liver fibrosis assessments.

In conclusion, we have shown in a well-characterised cohort of patients managed in a real-world setting, that curative HCV treatment halts fibrosis progression. The data support treatment for all people living with HCV, including people with mild-moderate liver fibrosis.

## Supporting information

S1 Fig A. Fibrosis staging of HCV cohort at baseline and follow-up (n = 114). B. Sustained virological response is associated with stable / improved liver fibrosis (n = 114). C. Persistent HCV is associated with liver fibrosis progression (n = 114).
(TIFF)

S1 Table Predictors of fibrosis progression (F0-F3 vs. F4). (DOCX)

S2 Table Predictors of liver fibrosis progression in HCV treatment failure. (DOCX)

S3 Table Predictors of fibrosis progression—Analysis of non-SVR and untreated patients. (DOCX)
