# Supplementary figures and images for: Sustained virological response halts fibrosis progression: A long-term follow-up study of people with chronic hepatitis C infection
Source: PLoS One. 2017 Oct 24;12(10):e0185609. doi: 10.1371/journal.pone.0185609 (PMC5655473; doi:10.1371/journal.pone.0185609)

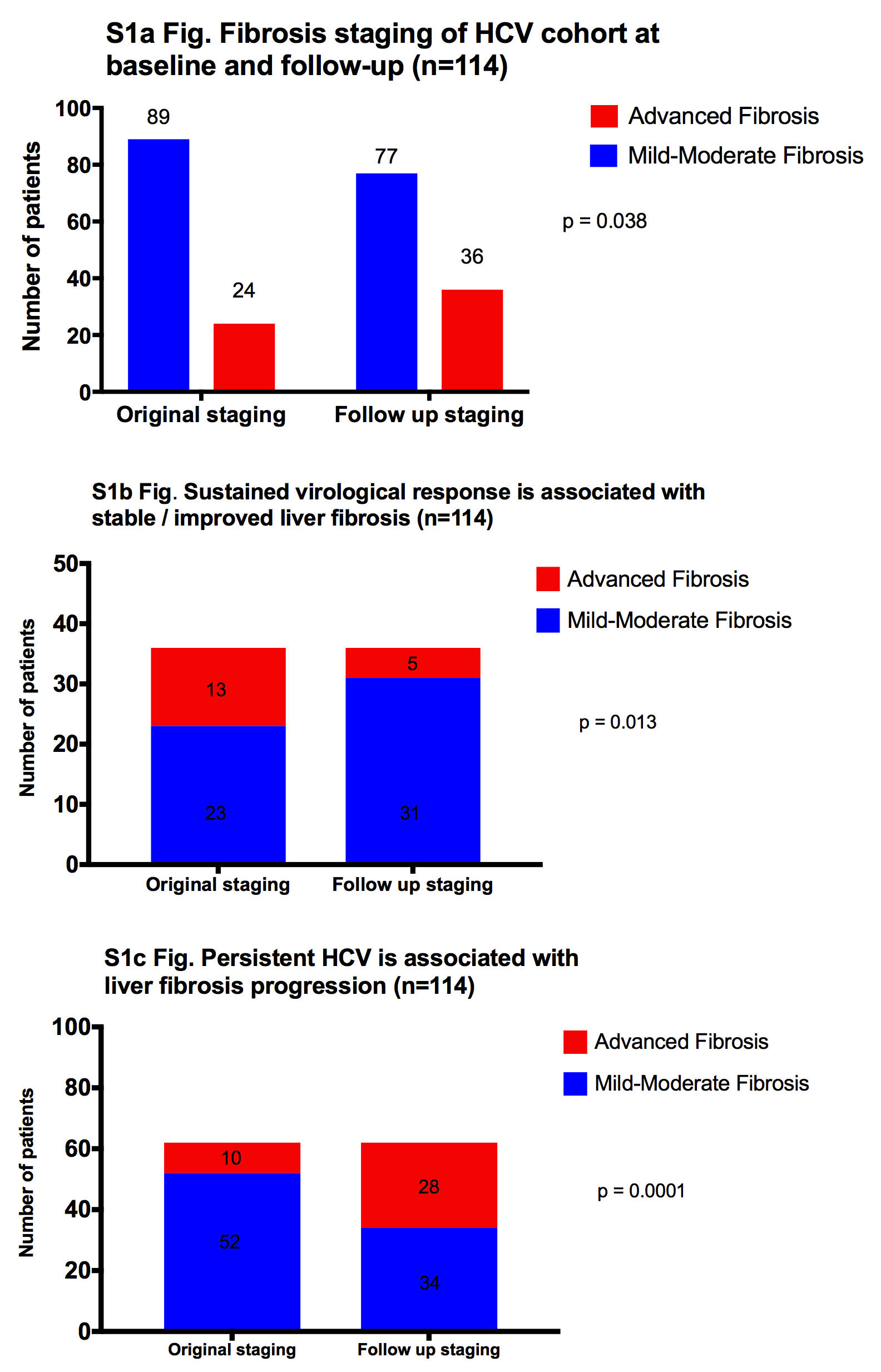

Supplement: S1 Fig — A. Fibrosis staging of HCV cohort at baseline and follow-up (n = 114). B. Sustained virological response is associated with stable / improved liver fibrosis (n = 114). C. Persistent HCV is associated with liver fibrosis progression (n = 114). (TIFF) [file pone.0185609.s001.tiff]
