# Supplementary material for: Sustained virological response halts fibrosis progression: A long-term follow-up study of people with chronic hepatitis C infection
Source: PLoS One. 2017 Oct 24;12(10):e0185609. doi: 10.1371/journal.pone.0185609 (PMC5655473; doi:10.1371/journal.pone.0185609)
Supplement: S1 Table — (DOCX) [file pone.0185609.s002.docx]

**S1 Table. Predictors of Fibrosis Progression (F0-F3 vs. F4)**

| **Co-variate** | **No Fibrosis progression** | **Fibrosis progression** | ***P*-value** | **Mutlivariate logistic regression (*P*-value)** |
| --- | --- | --- | --- | --- |
| *n* | 87 | 27 |  |  |
| Age of patient (yrs) | 57 (IQR 54-62) | 60 (IQR 57-63) | **0.072** |  |
| Gender (Male) | 61 (70%) | 18 (67%) | 0.638 |  |
| HCV genotype (G1 vs. other) | 53 (61%) | 16 (59%) | 0.366 |  |
| HCV acquisition (blood transfusion vs. other) | 19 (22%) | 12 (44%) | **0.051** |  |
| Estimated duration of HCV infection till liver biopsy | 16 (IQR 12.0-20) | 19.0 (IQR 15.0-27.0) | **0.015** |  |
| Fibrosis rate to original liver biopsy (METAVIR stage/years of infection) | 0.0488 (IQR 0-0.091) | 0.0253 (IQR 0-0.080) | 0.542 |  |
| Age of acquisition | 20 (IQR 17-22) | 21 (IQR 15-25) | 0.739 |  |
| Failure to attain SVR | 40 (46%) | 23 (85%) | **0.003** | **0.001** |
| Baseline ALT (U/L) | 86 (IQR 55-122) | 125(IQR 76-175) | **0.009** |  |
| Baseline Viral Load (IU/mL) | 859390 | 1.455log | 0.192 |  |
| Baseline ferritin | 218 (IQR 133-353) | 331 (IQR 162-631) | **0.044** |  |
| Baseline AFP |  |  | 0.141 |  |
| Caucasian vs. other | 71 (82%) | 24 (89%) | 1.000 |  |
| Estimated duration of infection till HCV treatment (yrs) | 24.0 (IQR 15.0-29.0) | 28.5(IQR 24.0 – 34.0) | **0.046** | **0.020** |
