# Supplementary material for: Sustained virological response halts fibrosis progression: A long-term follow-up study of people with chronic hepatitis C infection
Source: PLoS One. 2017 Oct 24;12(10):e0185609. doi: 10.1371/journal.pone.0185609 (PMC5655473; doi:10.1371/journal.pone.0185609)
Supplement: S2 Table — (DOCX) [file pone.0185609.s003.docx]

**S2 Table. Predictors of liver fibrosis progression in HCV treatment failure**

| **Co-variate** | **No Fibrosis progression** | **Fibrosis progression** | ***P*-value** | **Mutlivariate logistic regression (*P*-value)** |
| --- | --- | --- | --- | --- |
| *N* | 35 | 22 |  |  |
| Age of patient (yrs) | 56.0 (IQR 54-62) | 59.5 (IQR 56-64) | 0.062 |  |
| Gender (male) | 26 (74%) | 15 (68%) | 0.763 |  |
| HCV genotype (G1 vs. other) | 29 (83%) | 13 (59%) | 0.066 |  |
| HCV acquisition (blood transfusion vs. other) | 8 (23%) | 12 (55%) | **0.023** |  |
| Estimated duration of HCV infection till liver biopsy (yrs) | 16 (IQR 14-20) | 18 (IQR 15-28) | 0.225 |  |
| Age of acquisition | 20 (IQR 18-22) | 21 (IQR 16-26) | 0.763 |  |
| Baseline ALT (U/L) | 94 (IQR 59-127) | 133  (IQR 76-191) | 0.068 |  |
| Post-treatment ALT (U/L) | 50 (IQR 34-102) | 86 (IQR 54-130) | 0.139 |  |
| Baseline Viral Load (IU/mL) | 887858 (IQR 412775 – 2373705) | 1433720 (IQR 651705 – 1639510) | 0.681 |  |
| Baseline ferritin | 244 (IQR 112-334) | 417 (IQR 177-641) | **0.024** |  |
| Baseline AFP | 5 (IQR 3-6) | 6 (IQR 2-8) | 0.464 |  |
| Caucasian vs. other | 30 (86%) | 20 (91%) | 0.695 |  |
| Estimated duration of infection till HCV treatment (yrs) | 18.0 (IQR 15.0-25.0) | 28 (IQR 24.0 – 31) | **0.0001** | **0.0002** |
