# Supplementary material for: Sustained virological response halts fibrosis progression: A long-term follow-up study of people with chronic hepatitis C infection
Source: PLoS One. 2017 Oct 24;12(10):e0185609. doi: 10.1371/journal.pone.0185609 (PMC5655473; doi:10.1371/journal.pone.0185609)
Supplement: S3 Table — (DOCX) [file pone.0185609.s004.docx]

**S3 Table. Predictors of fibrosis progression – Analysis of non-SVR and untreated patients**

| **Co-variate** | **No Fibrosis progression** | **Fibrosis progression** | ***P*-value** | **Mutlivariate logistic regression (*P*-value)** |
| --- | --- | --- | --- | --- |
| *N* | 48 | 25 |  |  |
| Age of patient (yrs) | 57 (IQR 53-62) | 60 (IQR 56-63) | **0.090** |  |
| Gender (male) | 35 (73%) | 16 (64%) | 0.436 |  |
| HCV genotype (G1 vs. other) | 37 (77%) | 16 (64%) | 0.174 |  |
| HCV acquisition (blood transfusion vs. other) | 9 (19%) | 12 (48%) | **0.014** |  |
| Estimated duration of HCV infection till liver biopsy | 16 (IQR 12-20) | 18.5 (IQR 15-27) | 0.118 |  |
| Fibrosis rate to original liver biopsy (METAVIR stage/years of infection) | 0.028 (IQR 0-0.062) | 0 (IQR 0-0.072) | 0.867 |  |
| Age of acquisition | 20 (IQR 18-21) | 21 (IQR 16-26) | 0.365 |  |
| Baseline ALT (U/L) | 76 (IQR 55-109) | 129 (IQR 75-180) | **0.007** |  |
| Baseline Viral Load (IU/mL) | 859390(IQR 350245-1.89 log) | 1.43 log (IQR 909988– 6.68 log) | 0.331 |  |
| Baseline ferritin | 236 (IQR 132-320) | 374 (IQR 165-630) | **0.023** |  |
| Baseline AFP | 5 (IQR 4-8) | 6 (IQR 2-8) | 0.987 |  |
| Caucasian vs. other | 39 ( | 22 (88%) | 0.523 |  |
| Estimated duration of infection till HCV treatment (years) | 18.0 (IQR 15.0-25.5) | 27.5 (IQR 24.0 – 30.0) | **0.008** | **0.032** |
